# Association between renal-limited vasculitis and relapse of antineutrophil cytoplasmic antibody-associated vasculitis: A single-center retrospective cohort study in Japan

**DOI:** 10.1371/journal.pone.0274483

**Published:** 2022-09-29

**Authors:** Makoto Yamaguchi, Mayumi Ito, Hirokazu Sugiyama, Shiho Iwagaitsu, Hironobu Nobata, Hiroshi Kinashi, Takayuki Katsuno, Masahiko Ando, Yoko Kubo, Shogo Banno, Yasuhiko Ito, Takuji Ishimoto

**Affiliations:** 1 Department of Nephrology and Rheumatology, Aichi Medical University, Nagakute, Aichi, Japan; 2 Department of Nephrology and Rheumatology, Aichi Medical University Medical Center, Okazaki, Aichi, Japan; 3 Data Coordinating Center, Department of Advanced Medicine, Nagoya University Hospital, Nagoya, Japan; 4 Department of Preventive Medicine, Nagoya University Graduate School of Medicine, Nagoya, Japan; Nippon Medical School, JAPAN

## Abstract

**Background:**

Several previous studies have evaluated the predictors of relapse in antineutrophil cytoplasmic antibody-associated vasculitis. Nonetheless, the association between renal-limited vasculitis and relapse has not been evaluated.

**Objective:**

To assess the association between renal-limited vasculitis and the incidence of relapse in Japan among patients with microscopic polyangiitis/renal-limited vasculitis.

**Methods:**

This retrospective cohort study included consecutive patients in remission at 6 months, with renal-limited vasculitis (n = 24, renal-limited vasculitis group) and microscopic polyangiitis with renal and extra-renal involvement (n = 56, non-renal-limited vasculitis group) between 2004 and 2020.

**Results:**

During the median follow-up period of 35 (range, 15‒57) months, 28 (35.0%) patients had a relapse. Multivariable Cox proportional hazards models revealed that the lower estimated glomerular filtration rate (per -10 mL/min/1.73 m^2^; adjusted hazard ratio = 0.87, 95% confidence interval: 0.76–0.99; *P* =  0.043), renal-limited vasculitis (adjusted hazard ratio =  0.23, 95% confidence interval: 0.08–0.68; *P* =  0.008), and glucocorticoid combined with intravenous cyclophosphamide or rituximab (adjusted HR = 0.32, 95% CI: 0.11–0.96; *P* = 0.042) were associated with a decreased risk of relapse. Glucocorticoid dose during the observation period was lower in the renal-limited vasculitis group than in the non-renal-limited vasculitis group.

**Conclusions:**

Renal-limited vasculitis was associated with a lower risk of relapse than non-renal-limited vasculitis. Our data may contribute to the development of optimal management for renal-limited vasculitis, which may assist in minimizing the adverse effects of immunosuppressive therapy.

## Introduction

Antineutrophil cytoplasmic antibody (ANCA)-associated vasculitis (AAV) is a group of pauci-immune small-vessel vasculitides [1, 2]. AAV may be further subdivided according to its clinical and pathologic features into microscopic polyangiitis (MPA), granulomatosis with polyangiitis (GPA), eosinophilic granulomatosis with polyangiitis (EGPA), and renal-limited vasculitis (RLV) [1].

The majority of patients achieve and maintain remission through a combination of high-dose glucocorticoids and immunosuppressive drugs; nonetheless, relapses are common. Relapses are an important clinical issue in AAV because these require intensive immunosuppressive therapy and are associated with an increased risk of severe adverse events [3–6]. Although it is important to identify modifiable risk factors that can predict relapse, this remains undetermined and needs to be investigated.

MPA and GPA typically present with renal involvement [7]; however, the diagnosis is often delayed until advanced renal failure occurs. Advanced renal failure is associated with an increased risk of end-stage renal disease (ESRD) and death [8–10]. RLV is a subtype of AAV that presents with purely renal involvement. The renal manifestations of RLV may range from rapidly progressive glomerulonephritis (GN), such as pauci-immune necrotizing crescentic GN, to less aggressive but indolent disease that slowly progresses towards ESRD [11]. Although MPO-ANCA-positive MPA/RLV is the most common component of AAV in the Japanese population [12–14], little information about the association between RLV and incidence of relapse is available.

The present retrospective cohort study aimed to assess the association between RLV and the incidence of relapse in Japan among patients with MPA combined with renal and extra-renal involvement/RLV. The results of the present study provide pivotal information for the determination of the clinical goals of RLV treatments.

## Materials and methods

### Patients

**Fig 1:**
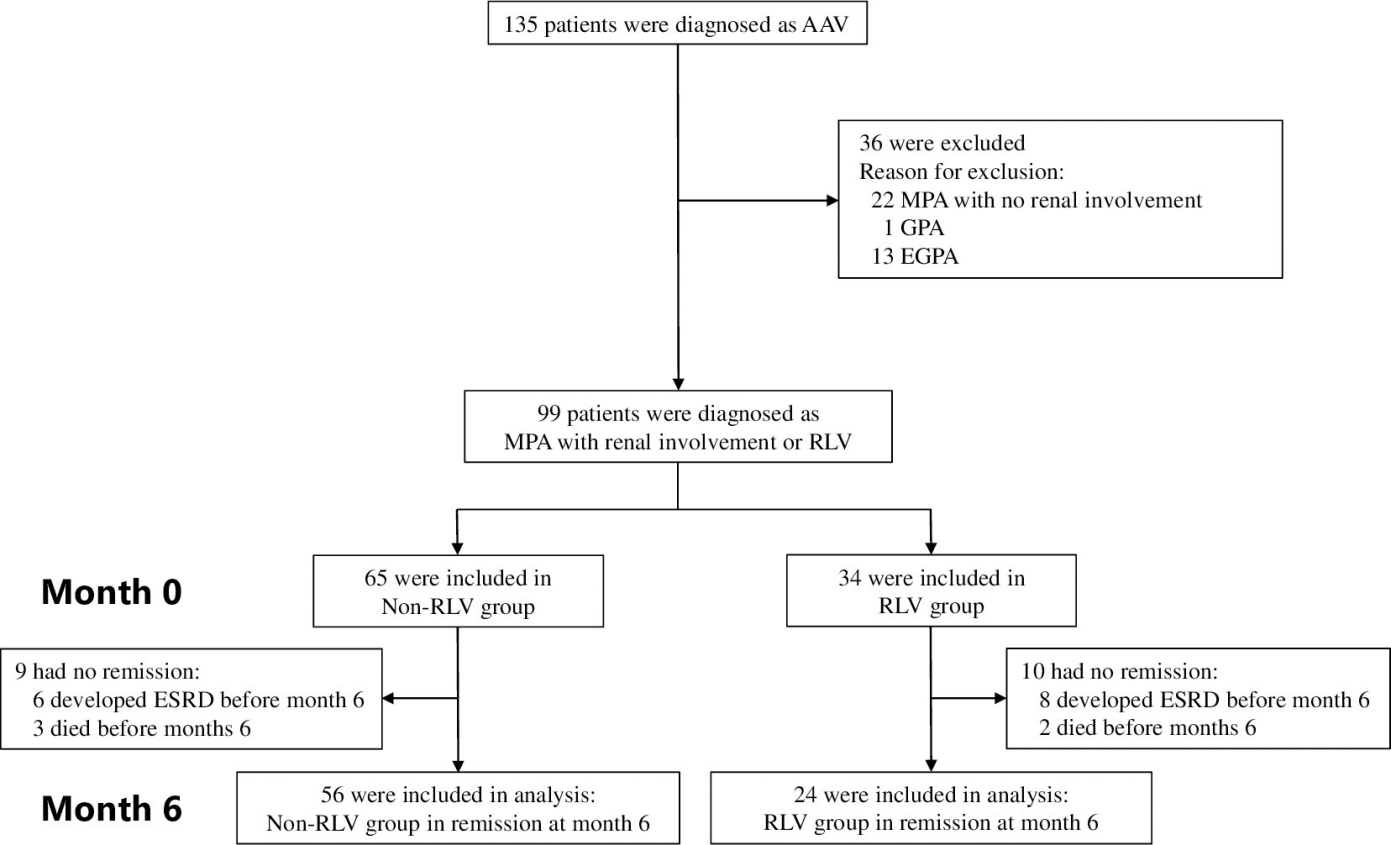
Flow diagram of patient selection. AAV, antineutrophil cytoplasmic autoantibody–associated vasculitis; EGPA, eosinophilic granulomatosis with polyangiitis; GPA, granulomatosis with polyangiitis; MPA, microscopic polyangiitis; RLV, renal-limited vasculitis.

A total of 135 adult patients were diagnosed with AAV at the Nephrology and Rheumatology centers of Aichi Medical University in Japan between 2004 and 2020. The diagnosis of AAV was made based on the criteria set by the European Medicines Agency [15]. After excluding 36 (26.7%) patients who were diagnosed with GPA (n = 1) or EGPA (n = 13), and did not have renal involvement (n = 22), 34 and 65 patients were diagnosed with RLV (RLV group), and MPA who had renal and extra-renal involvement (non-RLV group), respectively. In the RLV group, 10 patients did not achieve remission, eight developed ESRD and two died before 6 months; meanwhile, in the non-RLV group, nine patients did not achieve remission, six developed ESRD and three died before 6 months. Finally, 24 RLV and 56 non-RLV patients in remission at 6-month were included in the analysis (Fig 1). Remission was defined as BVAS of 0 at 6 months according to the European Alliance of Associations for Rheumatology recommendations for conducting clinical studies and/or clinical trials in systemic vasculitis [16].

### Procedures

This study was conducted in accordance with the ethical standards laid down in the World Medical Association’s Declaration of Helsinki. The study protocol was approved by the Ethics Committee of Aichi Medical University (approval number 2018-H350, November 3, 2019). The same committee waived the requirement for the acquisition of informed consent from patients owing to the retrospective nature of this study.

The following clinical characteristics were measured at the time of diagnosis of AAV and retrospectively collected from medical records, as previously described [17, 18]: age, sex, albumin, C-reactive protein levels, and estimated glomerular filtration rate (eGFR), Birmingham Vasculitis Activity Score (BVAS) 2003 [19] (general, cutaneous, ear, nose, throat, chest, cardiovascular, abdominal, renal, and nervous system involvement), vascular damage index (VDI) [19], renal biopsy findings, AAV GN classification (focal, mixed, cellular, and sclerotic), and severity of tubular atrophy and interstitial fibrosis (0%–5%, absent; ∔1, mild or 6%–24%; ∔2, moderate or 25%–49%; and ∔3, severe or ≥50%), as in a previous study [19]. Anti-myeloperoxidase and anti-proteinase 3 antibody levels were analyzed using direct antigen-specific enzyme-linked immunosorbent assays (Nipro Medical Corporation, Osaka, Japan) or 1:101 serial serum dilutions (Medical and Biological Laboratories Co., Ltd., Nagoya, Japan), as previously described [17, 18]. Immunosuppressive therapy data, such as whether induction was initiated with methylprednisolone pulse therapy (0.5 or 1.0 g/day for 3 consecutive days) or intravenous cyclophosphamide (IVCY) or rituximab (RTX), and whether maintenance therapy was continued with azathioprine (AZA), oral CY, methotrexate, and/or RTX, were documented. The point dose of prednisolone (PSL) (mg/day), cumulative dose of PSL (g), and use of any other immunosuppressants at 3, 6, 12, and 24 months after diagnosis were also noted.

### Main outcome variable

The primary outcome was the time from remission to disease relapse of AAV, which was defined as an increase in the BVAS from the previous visit that required an increase in the glucocorticoid dose and/or add-on use of immunosuppressive agents after a period of remission [20, 21]. Major relapse was defined as a relapse with organ-threatening or life-threatening disease activity [16].

Data on other outcomes, including eGFR 30% decrease, ESRD requiring dialysis, adverse events (hospitalization for infection, cardiovascular disease, bone fracture/femur head necrosis, diabetes mellitus, psychosis), or death, were also collected. The observation period was defined as the period from a time point 6 months after diagnosis to the incidence of relapse or the last follow-up in April 2022, whichever came first. In event of death before relapse, data was censored.

### Statistical analyses

The patients were divided into the RLV and non-RLV groups. Categorical variables are presented as percentages, whereas continuous variables are presented as medians with interquartile ranges (IQRs) for both normally and non-normally distributed data. The association between the non-time dependent covariates and primary outcomes were assessed using the univariable and multivariable Cox proportional hazards models.

The multivariable models were adjusted for clinically relevant confounding factors, such as age, sex, eGFR, induction immunosuppressive therapy (IVCY or RTX), and RLV. Furthermore, to confirm the robustness of the analysis, univariable binary logistic regression analysis for relapse was performed. Subsequently parameters with *P*-value <0.05 were included as covariates in the multivariable Cox proportional hazards models. The proportional hazard assumptions for the covariates were tested with scaled Schoenfeld residuals. The Kruskal–Wallis test was used to evaluate the significance of intergroup differences between continuous variables, whereas the Pearson’s chi-square test was used to compare categorical variables. To calculate relapse after remission, 80 patients who achieved remission at 6-month and were followed up thereafter were included. The cumulative probability of relapse was calculated using the Kaplan–Meier and log-rank tests.

The level of statistical significance was set at *P*<0.05. All statistical analyses were performed using JMP version 14.0.0 (SAS Institute, Cary, North Carolina, USA), SAS statistical software version 9.4 (SAS Institute), and STATA version 13.0 (StataCorp LP, College Station, Texas, USA).

## Results

### Clinical characteristics

The clinical characteristics of the RLV (n = 24) and non-RLV (n = 56) groups at the time of diagnosis are shown in Table 1. The CRP level and BVAS were significantly lower in the RLV group (*P* = 0.010, and 0.010, respectively). There were no significant differences in the other clinical characteristics between the two groups.

**Table 1:** Clinical characteristics of 80 patients in RLV and non-RLV groups who had achieved remission at 6 months.

	Non-RLV (n = 56)	RLV (n = 24)	*P* value
**Clinical characteristics**			
Age (year)	76 (70–78)	74 (68–81)	0.810
Male sex	30 (53.6)	13 (54.2)	0.961
eGFR (mL/min/1.73 m^2^)			
At diagnosis	34.2 (19.2–51.8)	29.6 (17.3–53.8)	0.629
At 6 months (at inclusion)	43.0 (24.2–59.9)	36.5 (22.3–58.8)	0.592
Serum albumin level (mg/dL)			
At diagnosis	2.9 (2.5–3.3)	3.1 (2.5–3.7)	0.478
At 6 months (at inclusion)	3.3 (2.9–3.8)	3.5 (3.0–4.0)	0.528
CRP level (mg/dL)			
At diagnosis	4.0 (1.7–8.4)	1.3 (0.4–3.1)	0.001
At 6 months (at inclusion)	0.1 (0.04–0.1)	0.1 (0.03–0.1)	0.759
Antibody			0.124
MPO-ANCA	56 (100)	23 (95.8)	
PR3-ANCA	0 (0)	1 (4.2)	
BVAS at diagnosis	18 (14–20)	14 (11–17)	0.010
General	56 (100)	22 (91.7)	0.029
Cutaneous	2 (3.6)	0 (0)	0.348
Ear, nose, and throat	17 (30.4)	0 (0)	0.002
Chest	31 (55.4)	0 (0)	<0.001
Cardiovascular	0 (0)	0 (0)	1.000
Abdominal	0 (0)	0 (0)	1.000
Renal	56 (100)	24 (100)	1.000
Nervous system	23 (41.1)	0 (0)	<0.001
VDI at diagnosis	2.8 (1.1–3.8)	1.8 (0.8–2.2)	0.014
Renal biopsy findings	n = 47	n = 22	
Number of glomeruli	12 (10–13)	11 (10–13)	0.489
AAV GN classification			0.893
Focal	13 (27.7)	5 (22.7)	
Mixed	18 (38.3)	8 (36.4)	
Cellular	13 (27.7)	8 (36.4)	
Sclerotic	3 (6.4)	1 (4.6)	
Tubular atrophy or interstitial fibrosis			0.667
Absent	2 (4.3)	2 (9.1)	
1∔	21 (44.7)	7 (31.8)	
2∔	19 (40.4)	11 (50.0)	
3∔	5 (10.6)	2 (9.1)	
Induction immunosuppressive therapy			
Glucocorticoid monotherapy	39 (69.6)	21 (86.1)	0.091
Intravenous cyclophosphamide	7 (12.5)	0 (0)	0.070
Rituximab	11 (19.6)	4 (16.7)	0.755
Use of mPSL pulse therapy	34 (60.7)	11 (45.8)	0.219
Maintenance immunosuppressive therapy			0.683
Glucocorticoid monotherapy	33 (58.9)	18 (75.0)	
Oral cyclophosphamide	2 (3.5)	0 (0)	
Azathioprine	10 (17.9)	2 (8.3)	
Methotrexate	1 (1.8)	0 (0)	
Mizoribine	4 (7.1)	2 (8.3)	
Rituximab	6 (3.5)	2 (8.3)	

^a^Continuous data are presented as medians (interquartile range), and categorical data are expressed as numbers (proportion).

^b^Abbreviations: eGFR, estimated glomerular filtration rate; CRP, C-Reactive Protein; MPO, myeloperoxidase; PR3, proteinase-3 ANCA; ANCA, antineutrophil cytoplasmic antibody; AAV, antineutrophil cytoplasmic antibody-associated vasculitis; BVAS, Birmingham Vasculitis Activity Score; VDI, vascular damage index; GN, glomerulonephritis; MPA, microscopic polyangiitis; RLV, renal-limited vasculitis; mPSL, methylprednisolone

### RLV and relapse

**Fig 2:**
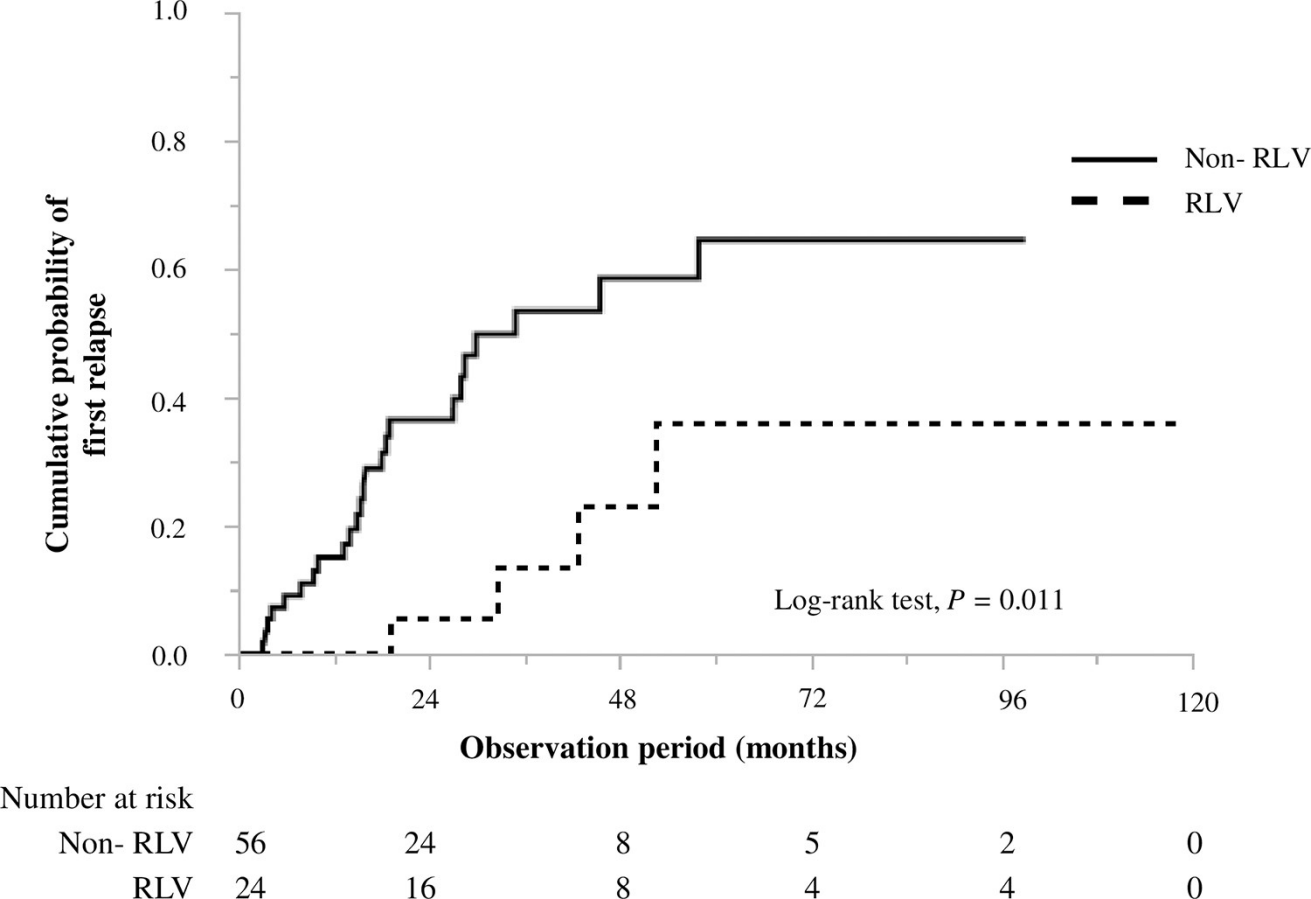
Cumulative probability of relapse in RLV and non-RLV groups. A significant difference was observed (*P* = 0.011). RLV, renal-limited vasculitis.

During the median follow-up period of 35 (range, 15‒57) months, four (16.7%) and 24 (42.9%) patients in the RLV and non-RLV groups, respectively, relapsed (*P* = 0.024, Table 2). Of the 28 relapse patients, one (25.0%) and 16 (66.7%) patients in the RLV and non-RLV groups, respectively, had a major relapse (*P* = 0.077). The cumulative probabilities of relapse within 1, 2, and 5 years of diagnosis were 0.00, 0.06, and 0.36 in the RLV group, respectively, and 0.15, 0.36, and 0.65 in the non-RLV group, respectively. Therefore, patients in the RLV group were at a lower risk of relapse than those in the non-RLV group (*P* = 0.011; Fig 2).

**Table 2:** Comparison of outcome data between RLV and non-RLV groups.

	Non-RLV (n = 56)	RLV (n = 24)	*P* value
**Outcome**			
Relapse	24 (42.9)	4 (16.7)	0.024
Major relapse	16 (66.7)	1 (25.0)	0.077
General	20 (83.3)	4 (100)	0.378
Cutaneous	0 (0)	0 (0)	1.000
Ear, nose, and throat	1 (4.2)	0 (0)	0.678
Chest	3 (12.5)	0 (0)	0.454
Cardiovascular	0 (0)	0 (0)	1.000
Abdominal	0 (0)	0 (0)	1.000
Renal	9 (37.5)	1 (25.0)	0.787
Nervous system	4 (16.7)	0 (0)	0.378
eGFR 30% decrease	9 (17.9)	4 (16.7)	0.812
ESRD	6 (10.7)	2 (8.3)	0.745
Death	8 (14.3)	2 (8.3)	0.287
Infection	4 (50.0)	1 (50.0)	
Vasculitis	1 (12.5)	0 (0)	
Malignancy	1 (12.5)	0 (0)	
Cardiovascular disease	0 (0)	1 (50.0)	
Unknown	1 (12.5)	0 (0)	
Adverse events			
Hospitalization for infection	14 (25.0)	5 (20.8)	0.688
Cardiovascular disease	2 (3.6)	2 (8.3)	0.371
Bone fracture/femur head necrosis	4 (7.1)	2 (8.3)	0.853
Diabetes mellitus	7 (12.5)	1 (4.2)	0.255
Psychosis	0 (0)	1 (4.2)	0.124
Observation period (months)	35 (14–58)	36 (19–56)	0.717

^a^Continuous data are presented as medians (interquartile range), and categorical data are expressed as numbers (proportion).

^b^Abbreviations: eGFR, estimated glomerular filtration rate; ESRD, end-stage renal disease

Among the patients who relapsed, renal involvement was documented in one (25.0%) and 9 (37.5%) patients in the RLV and non-RLV groups, respectively. There was no significant difference in the PSL dose between the RLV and non-RLV groups during relapse treatment (5 mg/day [IQR, 3–7] vs. 7 mg/day [IQR, 6–9]; *P* = 0.366), but the glucocorticoid dose was increased and/or an immunosuppressive agent was changed in all patients. In particular, the PSL dose in the RLV group was increased from 5 mg/day (IQR, 3–7) to 25 mg/day (IQR, 20–30), whereas the PSL dose in the non-RLV group was increased from 7 mg/day (IQR, 6–9) to 25 mg/day (IQR, 23–30). AZA, IVCY, and RTX were added to the immunosuppression regimens of two, one, and three patients, respectively. Following treatment, all patients achieved remission.

### Renal biopsy findings

A renal biopsy was performed in 22 (91.7%) and 47 (83.9%) patients in the RLV and non-RLV groups, respectively. Two-thirds of the patients in both groups demonstrated mixed and cellular vasculitis, and there were no significant differences in the classification of ANCA GN and severity of tubular atrophy and interstitial fibrosis between the two groups.

### Predictors of relapse

Patient background and treatment among the remission cases were compared between the patients with and without relapse (S1 Table). Age, proportion of RLV, and use of rituximab were higher among the patients without relapse, meanwhile, CRP level, BVAS, proportion of eye/nose/throat involvement, proportion of cellular type cases evaluated by renal biopsy, and VDI were higher among the patients with relapse.

According to a previous report [17–19, 22], the predictors of relapse included age, sex, eGFR, induction immunosuppressive therapy, and RLV. Univariable analyses demonstrated that lower eGFR values, RLV, and glucocorticoid combined with IVCY or RTX were associated with a decreased risk of relapse (Table 3). Multivariable analyses also confirmed that lower eGFR values (per -10 mL/min/1.73 m^2^; adjusted hazard ratio [HR] = 0.87, 95% confidence interval [CI]: 0.76–0.99; *P* =  0.043), RLV (vs. non-RLV, adjusted HR = 0.23, 95% CI: 0.08–0.68; *P* = 0.008), and glucocorticoid combined with IVCY or RTX (vs. glucocorticoid monotherapy, adjusted HR = 0.32, 95% CI: 0.11–0.96; *P* = 0.042) were associated with a decreased risk of relapse (Table 3).

**Table 3:** Predictors of the first relapse.

	Univariable model		Multivariable model	
	HR (95% CI)	*P*-value	HR (95% CI)	*P*-value
Age (per 10 years)	0.90 (0.70–1.20)	0.442	0.86 (0.66–1.16)	0.286
Male (vs. female)	0.85 (0.40–1.80)	0.679	0.79 (0.37–1.70)	0.553
eGFR (per –10 mL/min/1.73 m^2^)	0.85 (0.74–0.98)	0.024	0.87 (0.76–0.99)	0.043
Induction immunosuppressive therapy				
Glucocorticoid monotherapy	Reference		Reference	
Glucocorticoid + IVCY or RTX	0.45 (0.16–0.99)	0.048	0.32 (0.11–0.96)	0.042
RLV (vs. non-RLV)	0.27 (0.09–0.79)	0.017	0.23 (0.08–0.68)	0.008

^a^Data are presented as the HR, 95% CI, and *P* value from Cox proportional hazard regression analyses.

^b^“Glucocorticoid monotherapy” was used as the reference category.

^c^Data are adjusted for baseline characteristics, including age, sex, eGFR level, induction immunosuppressive therapy, and renal limited vasculitis.

^d^Abbreviations: HR, hazard ratio; CI, confidence interval; vs., versus; eGFR, estimated glomerular filtration rate; IVCY, intravenous cyclophosphamide; RTX, rituximab; RLV, renal-limited vasculitis

Furthermore, after performing a binary logistic regression analysis for relapse, age, immunosuppressive treatment, and RLV were identified, *P*-value <0.05 (S2 Table). After adjusting for these covariates in the multivariable Cox proportional hazard model, the results were not different from the original results (S3 Table), indicating a robust relationship between RLV and relapse.

### Immunosuppressive treatment during the observation period

The daily and cumulative doses of PSL at 0, 3, 6, 12, and 24 months after diagnosis were lower in the RLV group than in the non-RLV group. There were no significant differences in the use of other immunosuppressants at 3, 6, 12, and 24 months after diagnosis between the two groups (Table 4).

**Table 4:** Immunosuppressive treatment during the observation period.

	Non-RLV	RLV	*P*-value
At baseline (at diagnosis)	(n = 65)	(n = 34)	
Prednisolone (mg/day)	30 (30–40)	25 (20–40)	0.049
At the 3rd month	(n = 58)	(n = 28)	
Prednisolone (mg/day)	12 (10–15)	8 (7.5–10)	0.002
Cumulative dose of prednisolone (g)	1.6 (1.4–1.8)	1.3 (0.9–1.5)	<0.001
Use of immunosuppressants, n (%)	8 (13.7)	4 (14.3)	0.785
At the 6th month (at remission)	(n = 56)	(n = 24)	
Prednisolone (mg/day)	8 (7–10)	5 (5–7)	<0.001
Cumulative dose of prednisolone (g)	2.4 (2.0–2.9)	1.9 (1.5–2.3)	<0.001
Use of immunosuppressants, n (%)	12 (21.4)	4 (16.7)	0.695
At the 1st year	(n = 50)	(n = 12)	
Prednisolone (mg/day)	6 (5–8)	3 (3–5)	<0.001
Cumulative dose of prednisolone (g)	3.2 (2.7–3.7)	2.5 (2.1–3.0)	<0.001
Use of immunosuppressants, n (%)	10 (24.0)	4 (33.3)	0.362
At the 2nd year	(n = 24)	(n = 16)	
Prednisolone (mg/day)	5 (4–8)	2 (0–2)	<0.001
Cumulative dose of prednisolone (g)	4.3 (3.6–5.7)	3.6 (3.0–4.5)	0.019
Use of immunosuppressants, n (%)	15 (62.5)	4 (25.0)	0.081

^a^Continuous data are expressed as medians (interquartile range), and categorical data are expressed as numbers (proportion).

^b^Abbreviations: RLV, renal-limited vasculitis

### Other outcomes

During the observation period, eight (10.0%) patients developed ESRD requiring permanent dialysis therapy, which corresponded to two (8.3%) and six (10.7%) patients in the RLV and non-RLV groups, respectively (*P* = 0.745, Table 2). Hospitalization for infection was documented in five (20.8%) and 14 (25.0%) patients in the RLV and non-RLV groups, respectively (*P* = 0.688, Table 2). A total of 10 (12.5%) patients, which included two (8.3%) and eight (14.3%) patients in the RLV and non-RLV groups, respectively, died (*P* = 0.287, Table 2).

## Discussion

The present study showed that RLV was significantly associated with a lower risk of relapse than non-RLV (MPA patients with renal and extra-renal involvement); nevertheless, glucocorticoid dose was significantly lower in the RLV group than in the non-RLV group.

Two previous retrospective cohort studies examined the prognosis of RLV; however, their results were inconsistent [23, 24]. The first cohort study [23] examined 80 patients with AAV, including 20, 28, and 32 patients with RLV, MPA, and GPA, respectively, and reported lower incidences of ESRD and mortality in patients with RLV; however, the patients with RLV in this study had higher eGFR levels at baseline than those with MPA and GPA. Furthermore, this study did not examine whether immunosuppressive therapy contributed to the overall prognosis.

In contrast, the second cohort study [24] examined 502 patients with AAV, including 121, 264, and 117 patients with RLV, MPA, and GPA, respectively. This second study showed that compared with MPA and GPA, RLV was associated with an increased risk of a poor response to immunosuppressive therapy, ESRD, and mortality. While the relapse rate was lower in RLV than in MPA and GPA in this study, this study did not precisely evaluate renal function, other organ involvement, and immunosuppressive treatment, and its results should be interpreted with caution.

A previous study that investigated four randomized control trials (RCTs) on AAV examined the association between kidney function at baseline and the incidence of relapse [25]. This study demonstrated that higher baseline eGFR was a risk factor for relapse, and our data were compatible with this. This finding was paradoxical because it indicated that a more severe disease was associated with a lower risk of relapse; however, renal dysfunction results in some immunosuppression, which may explain this finding [26–29]. It must be noted that the majority of the patients in this study had GPA, and the proportion of patients with renal involvement was small (16.3%). It was unclear whether the study’s results included patients with MPA with renal involvement. In the present study, renal biopsy findings demonstrated that the sclerotic type showed lower eGFR levels than other types (P = 0.035, S4 Table). The sclerotic type might be considered to be a more irreversible state compared with the other types; namely, there might be a small number of normal glomeruli that could be lesions indicating renal relapse. This might be one a reason that lower eGFR was associated with a decreased risk of relapse. Further studies should be done to clarify these mechanisms.

In the present study, organ involvement of relapses in the RLV group demonstrated only renal and general symptoms. However, relapses in the non-RLV group were not only renal involvement but also non-renal organs, including the nervous system, eye/nose/throat, and lung lesions. Furthermore, all cases of relapse in non-RLV groups demonstrated organ damage that was the same as the primary ones at the initial presentation. These results indicated that relapse occurs differently among patients with RLV and non-RLV. Regarding monitoring for relapse, physicians should take care of not only renal involvement but also non-renal involvement among patients with non-RLV. It should be evaluated whether any relapsed organ was the same as that in the primary one in prospective cohort studies with larger sample size.

Our study also indicated no significant difference in the incidence of hospitalization for infection between the RLV and non-RLV groups, irrespective of the glucocorticoid dose in either group. Patients with RLV had a lower risk of relapse, and future research should investigate how to minimize the risk of severe infection in this population.

As for immunosuppressive treatment, our study demonstrated that induction treatment with glucocorticoid combined with RTX or IVCY was associated with a lower risk of relapse than that of glucocorticoid monotherapy; this was compatible with previous studies [30, 31]. However, in our study, glucocorticoid monotherapy, which is not the standard treatment worldwide, was frequently administered; as such, the results should be interpreted cautiously.

The present study has some limitations. First, this was a retrospective observational study, and we were not able to exclude all possible confounding factors. Second, our study examined patients with RLV and MPA with renal and extra-renal involvement; therefore, our results may not be generalizable to patients with other types of AAV, such as GPA. Third, we could not examine whether there was an indication bias for glucocorticoid monotherapy or combination therapy with RTX or IVCY, and treatment strategies were different between the two groups. Owing to the retrospective nature of this study; as such, it may be difficult to generalize the results. Fourth, our sample size was small, and our data should be validated in larger cohort studies.

In conclusion, the present study revealed that RLV was associated with a lower risk of relapse, as compared with non-RLV; RLV may have distinct clinical characteristics that distinguish it from non-RLV. Our data may contribute to the development of optimal management for RLV, which may assist in minimizing the adverse effects of immunosuppressive therapy.

## Supporting information

S1 Table Clinical characteristics of 80 patients with relapse and non-relapse. (DOCX)

S2 Table Univariate analysis in binary logistic regression to determine the predictors of relapse. (DOCX)

S3 Table Predictors of the first relapse. (DOCX)

S4 Table Clinical characteristics of 69 patients who underwent renal biopsy compared on the basis of histologic classification. (DOCX)

S1 Data Anonymized data set. (XLSX)
