# Supplementary material for: Association between renal-limited vasculitis and relapse of antineutrophil cytoplasmic antibody-associated vasculitis: A single-center retrospective cohort study in Japan
Source: PLoS One. 2022 Sep 29;17(9):e0274483. doi: 10.1371/journal.pone.0274483 (PMC9522015; doi:10.1371/journal.pone.0274483)
Supplement: S1 Table — (DOCX) [file pone.0274483.s001.docx]

**S1 Table.** Clinical characteristics of 80 patients with relapse and without relapse

|  | Relapse (-)  (n = 52) | Relapse (+)  (n = 28) | *P* value |
| --- | --- | --- | --- |
| **Clinical characteristics at diagnosis** |  |  |  |
| Age (year) | 77 (71–81) | 72 (66–77) | 0.012 |
| Male sex | 30 (57.7) | 13 (46.4) | 0.335 |
| eGFR (mL/min/1.73 m^2^) | 32.3 (15.6–47.9) | 37.3 (20.1–57.2) | 0.242 |
| Serum albumin level (mg/dL) | 2.9 (2.5–3.5) | 3.1 (2.5–3.5) | 0.964 |
| CRP level (mg/dL) | 2.2 (0.8–4.2) | 5.9 (3.1–9.9) | <0.001 |
| Antibody |  |  | 0.460 |
| MPO-ANCA | 51 (98.1) | 28 (100) |  |
| PR3-ANCA | 0 (0) | 1 (1.9) |  |
| BVAS | 14 (12–16) | 17 (14–18) | 0.043 |
| General | 50 (96.2) | 28 (100) | 0.293 |
| Cutaneous | 2 (3.9) | 0 (0) | 0.293 |
| Ear, nose, and throat | 7 (13.5) | 10 (35.7) | 0.020 |
| Chest | 21 (40.4) | 10 (35.7) | 0.566 |
| Cardiovascular | 0 (0) | 0 (0) | 1.000 |
| Abdominal | 0 (0) | 0 (0) | 1.000 |
| Renal | 56 (100) | 24 (100) | 1.000 |
| Renal-limited vasculitis | 20 (38.5) | 4 (14.3) | 0.024 |
| Nervous system | 15 (30.8) | 7 (25.0) | 0.587 |
| VDI | 2.0 (1.7–2.9) | 2.9 (1.6–3.9) | 0.012 |
| Renal biopsy findings | n = 47 | n = 22 |  |
| Number of glomeruli | 12 (10–13) | 11 (10–12) | 0.379 |
| AAV GN classification |  |  | <0.001 |
| Focal | 16 (34.0) | 2 (9.1) |  |
| Mixed | 21 (44.7) | 5 (22.7) |  |
| Cellular | 6 (12.8) | 15 (68.2) |  |
| Sclerotic | 4 (8.5) | 0 (0) |  |
| Tubular atrophy or interstitial fibrosis |  |  | 0.074 |
| Absent | 4 (8.5) | 0 (0) |  |
| 1∔ | 15 (31.9) | 13 (59.1) |  |
| 2∔ | 24 (51.1) | 6 (27.3) |  |
| 3∔ | 4 (8.5) | 3 (13.6) |  |
| Induction immunosuppressive therapy |  |  |  |
| Glucocorticoid monotherapy | 36 (69.2) | 25 (89.3) | 0.044 |
| Intravenous cyclophosphamide | 3 (5.8) | 4 (14.3) | 0.199 |
| Rituximab | 14 (26.9) | 1 (3.6) | 0.011 |
| Use of mPSL pulse therapy | 33 (63.5) | 12 (42.9) | 0.076 |
| Maintenance immunosuppressive therapy |  |  | 0.027 |
| Glucocorticoid monotherapy | 34 (65.4) | 17 (60.7) |  |
| Oral cyclophosphamide | 2 (3.9) | 0 (0) |  |
| Azathioprine | 4 (7.7) | 8 (28.6) |  |
| Methotrexate | 0 (0) | 1 (3.6) |  |
| Mizoribine | 4 (7.7) | 2 (7.1) |  |
| Rituximab | 8 (15.4) | 0 (0) |  |

^a^Continuous data are presented as medians (interquartile range), and categorical data are expressed as numbers (proportion).

^b^Abbreviations: eGFR, estimated glomerular filtration rate; CRP, C-Reactive Protein; MPO, myeloperoxidase; PR3, proteinase-3 ANCA; ANCA, antineutrophil cytoplasmic antibody; AAV, antineutrophil cytoplasmic antibody-associated vasculitis; BVAS, Birmingham Vasculitis Activity Score;
