# Supplementary material for: Association between renal-limited vasculitis and relapse of antineutrophil cytoplasmic antibody-associated vasculitis: A single-center retrospective cohort study in Japan
Source: PLoS One. 2022 Sep 29;17(9):e0274483. doi: 10.1371/journal.pone.0274483 (PMC9522015; doi:10.1371/journal.pone.0274483)
Supplement: S2 Table — (DOCX) [file pone.0274483.s002.docx]

**S2 Table.** Univariate analysis in binary logistic regression to determine the predictors of relapse

| Variable | OR (95% CI) | *P* value |
| --- | --- | --- |
| Age (per 10 years) | 0.61 (0.40–0.95) | 0.019 |
| Male sex (vs. female sex) | 0.64 (0.25–1.60) | 0.335 |
| eGFR (per -10 mL/min/1.73 m^2^) | 0.86 (0.72–1.04) | 0.114 |
| Serum albumin level (mg/dL) | 0.82 (0.14–4.81) | 0.828 |
| CRP level (per 1 mg/dL) | 1.06 (0.97–1.16) | 0.126 |
| Glucocorticoid + IVCY or RTX (vs. Glucocorticoid monotherapy) | 3.70 (1.00–14.1) | 0.034 |
| RLV (vs. non-RLV) | 3.75 (1.13–12..4) | 0.030 |

^a^Data are presented as the OR, 95% CI, and *P* value from binary logistic regression analysis.

^b^Abbreviations: OR, odds ratio; CI, confidence interval; vs., versus; eGFR, estimated glomerular filtration rate; CRP, C-Reactive Protein; IVCY, intravenous cyclophosphamide; RTX, rituximab; RLV, renal-limited vasculitis
