# Supplementary material for: Association between renal-limited vasculitis and relapse of antineutrophil cytoplasmic antibody-associated vasculitis: A single-center retrospective cohort study in Japan
Source: PLoS One. 2022 Sep 29;17(9):e0274483. doi: 10.1371/journal.pone.0274483 (PMC9522015; doi:10.1371/journal.pone.0274483)
Supplement: S3 Table — (DOCX) [file pone.0274483.s003.docx]

**S3 Table.** Predictors of the first relapse

|  | Multivariable model | |
| --- | --- | --- |
|  | HR (95% CI) | *P*-value |
| Age (per 10 years) | 0.88 (0.67–1.19) | 0.369 |
| Induction immunosuppressive therapy |  |  |
| Glucocorticoid monotherapy | Reference |  |
| Glucocorticoid + IVCY or RTX | 0.23 (0.07–0.78) | 0.019 |
| RLV (vs. non-RLV) | 0.22 (0.07–0.63) | 0.005 |

^a^Data are presented as the HR, 95% CI, and *P* value from Cox proportional hazard regression analyses.

^b^“Glucocorticoid monotherapy” was used as the reference category.

^c^Data are adjusted for baseline characteristics, including age, induction immunosuppressive therapy, and renal limited vasculitis.

^d^Abbreviations: HR, hazard ratio; CI, confidence interval; vs., versus; IVCY, intravenous cyclophosphamide; RTX, rituximab; RLV, renal-limited vasculitis
