# Supplementary material for: Association between renal-limited vasculitis and relapse of antineutrophil cytoplasmic antibody-associated vasculitis: A single-center retrospective cohort study in Japan
Source: PLoS One. 2022 Sep 29;17(9):e0274483. doi: 10.1371/journal.pone.0274483 (PMC9522015; doi:10.1371/journal.pone.0274483)
Supplement: S4 Table — (DOCX) [file pone.0274483.s004.docx]

**S4 Table.** Clinical characteristics of 69 patients who underwent renal biopsy compared on the basis of histologic classification

|  | Focal  (n=18) | Mixed  (n=26) | Crescentic  (n=21) | Sclerotic  (n=4) | *P* value |
| --- | --- | --- | --- | --- | --- |
| **Clinical characteristics** |  |  |  |  |  |
| Age (year) | 77 (73-82) | 77 (71-78) | 68 (54-79) | 78 (71-78) | 0.012 |
| Male sex | 11 (61.1) | 15 (57.7) | 10 (47.6) | 1 (25.0) | 0.512 |
| eGFR (mL/min/1.73m^2^) | 37 (25-46) | 31 (18-43) | 35 (22-66) | 11 (4-55) | 0.204 |
| RLV | 5 (27.8) | 8 (30.8) | 8 (38.1) | 1 (25.0) | 0.893 |
| Outcome |  |  |  |  |  |
| Relapse | 2 (11.1) | 5 (19.2) | 15 (71.4) | 0 (0) | <0.001 |
| ESRD | 0 (0) | 0 (0) | 2 (9.5) | 1 (25.0) | 0.059 |
| Death | 1 (5.6) | 2 (7.7) | 3 (14.3) | 2 (50.0) | 0.074 |

^a^Continuous data are presented as a median (interquartile range), and categorical data are expressed as a number (proportion).

^b^Abbreviations: eGFR, estimate glomerular filtration rate; RLV, renal-limited vasculitis; ESRD, end-stage renal disease
